# The Tol-Pal System of Uropathogenic *Escherichia coli* Is Responsible for Optimal Internalization Into and Aggregation Within Bladder Epithelial Cells, Colonization of the Urinary Tract of Mice, and Bacterial Motility

**DOI:** 10.3389/fmicb.2019.01827

**Published:** 2019-08-07

**Authors:** Hidetada Hirakawa, Kazutomo Suzue, Kumiko Kurabayashi, Haruyoshi Tomita

**Affiliations:** ^1^Department of Bacteriology, Graduate School of Medicine, Gunma University, Gunma, Japan; ^2^Department of Infectious Diseases and Host Defense, Graduate School of Medicine, Gunma University, Gunma, Japan; ^3^Laboratory of Bacterial Drug Resistance, Graduate School of Medicine, Gunma University, Gunma, Japan

**Keywords:** virulence, invasion, biofilm, pyelonephritis, urinary tract infection (UTI)

## Abstract

Urinary tracts infection (UTI) caused by uropathogenic *Escherichia coli* (UPEC) is a common infectious disease. With the shortage of new antimicrobial agents, the increase in UPEC resistance to commonly used drugs, such as fluoroquinolones and β-lactams including carbapenems is a critical issue. UPEC invades urinary tract cells, where it aggregates, and subsequently, forms biofilm-like multicellular colonies termed intracellular bacterial communities (IBCs). This process allows the bacteria to establish infections and so may be a good potential target for new drugs to treat infections. Here, we show that deletion of the *tolB* gene, encoding a protein of the Tol-Pal system that was originally characterized as a protein complex for colicin uptake and maintenance of the outer membrane, decreases the level of bacterial internalization into and aggregation within cultured bladder epithelial cells and also inhibits the colonization of mice urinary tracts. The *tolB* mutant also exhibited defective motility because of impaired flagellum syntheses. The *fliC* and *motA* mutants, which are non-motile strains, also exhibited lower levels of bacterial internalization and aggregation than their wild-type parent. Additional deletion of *tolB* in the *fliC* mutant did not further decrease these, suggesting that the attenuated virulence of the *tolB* mutant is a result of defective motility. The *tolA*, *tolQ*, *tolR*, and *pal* mutants that lack other members of the Tol-Pal system also exhibited lower levels of motility and aggregation within bladder epithelial cells compared to their wild-type parent. These combined results suggest another role of the Tol-Pal system, i.e., that it is responsible for optimal internalization, aggregation followed by IBC formation within urinary tract cells, and bacterial motility.

## Introduction

Urinary tract infection (UTI) is one of the most common infectious diseases. Uropathogenic *Escherichia coli* (UPEC) is a major pathogen that is estimated to cause over 80% of uncomplicated UTIs (Ronald, 2003; Zhang and Foxman, 2003).

When UPEC initially enters a urinary tract, the bacteria adhere to and invade the host bladder epithelial cells, where they aggregate and form biofilm-like multicellular microbial colonies termed intracellular bacterial communities (IBCs). IBCs protect UPEC from antimicrobial agents and the host immune system (Mulvey et al., 1998; Anderson et al., 2003). Therefore, bacterial adherence to, internalization into and aggregation within bladder epithelial cells followed by IBCs formation are key processes in determining whether infection becomes refractory to treatment.

Type 1 fimbriae are encoded by *fim* genes and are major fimbriae of this bacterium. They are required for initial attachment to and internalization into bladder epithelial cells, while motility contributes to bacterial fitness and migration to infection sites as the bacteria colonize the bladder (Martinez et al., 2000; Lane et al., 2005; Wright et al., 2005; Wright et al., 2007).

UPEC strains that have developed resistance to commonly used antimicrobials such as fluoroquinolones and β-lactams including carbapenems have been commonly isolated (Mediavilla et al., 2016; Zowawi et al., 2015). Therefore, new approaches are needed for the treatment of refractory infections, such as drugs that target proteins that are required for bacterial pathogenicity.

We have been interested in identifying genes that are responsible for adherence to, internalization into, and aggregation and/or IBCs formation within bladder epithelial cells. We carried out a genetic screen previously using a transposon library to identify genes that are required for bacterial adhesion and aggregation in a 96-well polystyrene plate. This assay termed static biofilm assay, is often used to evaluate adhesion and aggregation that may be associated with IBCs formation (O’Toole, 2011). During this process, we identified the *fur* gene as a repressor of adhesion to, internalization into and aggregation within bladder epithelial cells because the *fur* mutant exhibited a higher degree of adhesion and aggregation in a 96-well polystyrene plate (Kurabayashi et al., 2016). The mutant constitutively produced large numbers of both of type 1 fimbriae and flagella, which resulted in a higher rate of adhesion to, internalization into and aggregation within bladder epithelial cells than the wild-type parent. In contrast to *fur*, we also identified a strain that exhibited a lower level of adhesion and aggregation on a 96-well plate than the wild-type parent and found that in this case the transposon had been inserted into the *tolB* gene.

The *tolB* gene product is localized to the periplasmic space, where it interacts with TolA, TolQ, TolR, and Pal, forming two protein complexes that constitute the Tol-Pal system (Lazzaroni et al., 1999; Walburger et al., 2002). These proteins were originally characterized by their involvement in the uptake of group A colicins (Nagel de Zwaig, 1969; Webster, 1991). Additionally, these proteins contribute to outer-membrane stability by interacting with some outer-membrane localized proteins such as OmpA (Clavel et al., 1998). The *tolQ*, *tolR*, *tolA*, and *tolB* genes are transcribed with *ybgC*, which is the first gene composing the operon, while the *pal* gene is located downstream of *tolB* and is transcribed with *ybgF* as part of another operon (Vianney et al., 1996; Muller and Webster, 1997). Interestingly, the *ybgC* and *ybgF* gene products are not part of the Tol-Pal system.

In this study, we initially focused on the *tolB* gene, and characterized its role in adhesion to, internalization into and aggregation within the bladder epithelial cells. We found that the *tolB* mutant is internalized into and aggregates within the bladder epithelial cells with low efficiency, although it still adheres to the bladder epithelial cells at similar level to the wild-type parent. Viable cell numbers of the mutant in the bladder of mice were much lower than those of the wild-type parent. In addition to the *tolB* gene, we also investigated the role of other *tol*-*pal* genes together with the uncharacterized *ybgC* and *ybgF* genes. We found that *tolA*, *tolQ*, *tolR*, and *pal* mutants, but not *ybgC* and *ybgF* mutants, were moderately internalized into and aggregated within bladder epithelial cells similarly to the *tolB* mutant. We also show that the *tol*-*pal* genes are also required for optimal motility of UPEC, and that motility is responsible for bacterial internalization into, and aggregation within bladder epithelial cells.

## Materials and Methods

### Bacterial Strains, Host Cells and Culture Conditions

Table 1 lists the bacterial strains and plasmids used in this study. UPEC CFT073 (Welch et al., 2002) and its derivative strains were grown in Luria-Bertani (LB), RPMI1640 (Sigma-Aldrich, St. Louis, MO, United States) or artificial urine medium (AUM) (Brooks and Keevil, 1997). Cell growth was monitored at OD_600__._ Antibiotics were added to growth media for marker selection and maintaining plasmids at the following concentrations; ampicillin (150 μg/mL), chloramphenicol (45 μg/mL) and kanamycin (50 μg/mL). To heterologously express *tolB*, *fliC*, and *motA* from plasmids using isopropyl-β-D-thiogalactopyranoside (IPTG)-inducible promoter, 0.01 mM of IPTG were added in the culture media. In our preliminary complementation experiments with the bladder epithelial cells, bacterial internalization of the *tolB* mutant was complemented by introducing the *tolB* expression plasmid in the presence of 0.01 mM IPTG. On the other hand, no complementation was observed in the absence of IPTG while moderate growth defect was observed when 0.1 mM IPTG was added due to overexpression of its gene product. Therefore, we expected that 0.01 mM IPTG addition makes bacteria produce gene products at appropriate level in our system. HTB-9 bladder epithelial cells (Fogh, 1978) were cultured in RPMI1640 medium containing 10% HyClone FetalClone III serum (HyClone Laboratories, Inc., Logan, UT, United States) at 37°C and in an atmosphere of 5% CO_2_.

**TABLE 1:** Strains and plasmids used in this study.

**Strain or plasmid**	**Relevant genotype/phenotype**	**References**
**Strains**		
CFT073	Wild-type parent strain (ATCC 700928)	Welch et al., 2002
CFT073ΔtolB	*tolB* mutant from CFT073	This work
CFT073ΔtolA	*tolA* mutant from CFT073	This work
CFT073ΔtolQ	*tolQ* mutant from CFT073	This work
CFT073ΔtolR	*tolR* mutant from CFT073	This work
CFT073Δpal	*pal* mutant from CFT073	This work
CFT073ΔybgC	*ybgC* mutant from CFT073	This work
CFT073ΔybgF	*ybgF* mutant from CFT073	This work
CFT073ΔfliC	*fliC* mutant from CFT073	This work
CFT073ΔmotA	*motA* mutant from CFT073	This work
CFT073ΔompA	*ompA* mutant from CFT073	This work
CFT073ΔtolBΔfliC	*tolB* and *fliC* double mutant from CFT073	This work
**Plasmids**		
pKO3	Temperature sensitive vector for gene targeting, *sacB*, Cm^R^	Link et al., 1997
pTrc99K	Vector for IPTG-inducible expression; Km^R^	Hirakawa et al., 2003
pTrc99KtolB	*tolB* expression plasmid; Km^R^	This work
pTrc99KfliC	*fliC* expression plasmid; Km^R^	This work
pTrc99KmotA	*motA* expression plasmid; Km^R^	This work
pTurboGFP-B	GFP expression plasmid; Ap^R^	Evrogen

*Cm^*R*^, Chloramphenicol resistance; Km^*R*^, kanamycin resistance; Ap^*R*^, ampicillin resistance.*

### General DNA Manipulations

Polymerase chain reaction (PCR) was run using KOD FX Neo DNA polymerase by following the manufacturer’s instructions (TOYOBO, Osaka, Japan). Plasmids were introduced into *E. coli* by electroporation on MicroPulser (Bio-Rad Laboratories, Hercules, CA, United States). For DNA purification, we used PureLink Quick PCR purification kit and Wizard Plus SV Minipreps DNA Purification System by following the manufacturer’s instructions (Thermo Fisher Scientific, Waltham, MA, and Promega Corp., Madison, WI, United States, respectively). DNA sequencing was performed by a customer service of Eurofins Genomics (Tokyo, Japan).

### Cloning and Mutant Construction

To design primers, CFT073 genome sequence was obtained from a database in National Center for Biotechnology Information (NCBI) (Reference Sequence: NC_004431.1). In-frame gene deletions were produced by sequence overlap extension PCR using a strategy described previously (Link et al., 1997), and using the primer pairs, delta1/delta2 and delta3/delta4 primers, as shown in Table 2. The upstream flanking DNA included 450 bp and the first 2 amino acid codons of *tolB*, the first 3 amino acid codons of *pal*, *tolA*, *tolQ*, *tolR*, *ybgC*, *fliC*, *motA*, and *ompA*, and the first 4 amino acid codons of *ybgF*. The downstream flanking DNA included, the last 1 amino acid codon (CTG) of *tolB*, the last 5 amino acid codons of *pal*, the last 2 amino acid codons of *tolA*, *ybgF*, *fliC*, and *ompA*, the last 7 amino acid codons of *tolQ* and *ybgC*, the last 3 amino acid codons of *tolR*, the last 16 amino acid codons of *motA*, the stop codon, and 450 bp of DNA. These deletion constructs were ligated into the temperature sensitive vector pKO3 (Link et al., 1997) using *Bam*HI and *Sal*I digestion for *tolA*, *tolQ*, *tolR motA*, *ybgC*, *ybgF*, and *ompA*, *Not*I and Sal digestion for *tolB* and *pal*, or *Bam*HI and *Sma*I digestion for *fliC*, and introduced into CFT073, the wild-type parent strain. We selected sucrose-resistant/chloramphenicol-sensitive colonies at 30°C and confirmed the resulting deletion mutant strains using PCR analysis and DNA sequencing.

**TABLE 2:** Primers used in this study.

**Primer**	**DNA sequence (5′ – 3′)**	**Use**
tolB-delta1	gcggcggccgcgtgagctaagctctggtaag	*tolB* mutant construction
tolB -delta2	ctattcaattaattattatcacagcttcatcatatctcccttatc	*tolB* mutant construction
tolB -delta3	gataagggagatatgatgaagctgtgataataattaattgaatag	*tolB* mutant construction
tolB -delta4	gcggtcgacgtactgcaggtttttctttac	*tolB* mutant construction
tolA-delta1	gcgggatccaatcaacattgtaccgttgc	*tolA* mutant construction
tolA-delta2	agtcaacatcgcgattacggtttctttgacactctcggtttcc	*tolA* mutant construction
tolA-delta3	tttggaaaccgagagtgtcaaagaaaccgtaatcgcgatgttg	*tolA* mutant construction
tolA-delta4	gcggtcgacggagtgacctgaccgacaac	*tolA* mutant construction
tolQ-delta1	gcgggatccatagtagcagcgtttaaaagc	*tolQ* mutant construction
tolQ-delta2	taccccttgttgctctcgctaacgatattcatgtcagtcactg	*tolQ* mutant construction
tolQ-delta3	aagcagtgactgacatgaatatcgttagcgagagcaacaaggg	*tolQ* mutant construction
tolQ-delta4	gcggtcgacatgtttagataggctgcgtc	*tolQ* mutant construction
tolR-delta1	gcgggatcctttacagcggcttcaaagag	*tolR* mutant construction
tolR-delta2	aacgcagatgtttagataggctgtctggccatggcttacccc	*tolR* mutant construction
tolR-delta3	acaaggggtaagccatggccagacagcctatctaaacatctgc	*tolR* mutant construction
tolR-delta4	gcggtcgactcggcctgttttgcggcttc	*tolR* mutant construction
pal-delta1	gcggcggccgccgacctggttcccggacag	*pal* mutant construction
pal-delta2	ttctcttagtaaaccagtaccgccagttgcatttcaatgattcc	*pal* mutant construction
pal-delta3	aaggaatcattgaaatgcaactggcggtactggtttactaagag	*pal* mutant construction
pal-delta4	gcggtcgacgatttatcctgcaccagcgc	*pal* mutant construction
ybgC-delta1	gcgggatcctctgatgagtaagattatcg	*ybgC* mutant construction
ybgC-delta2	cactgcttaaactccgcgacaattgtattcactttacatcccg	*ybgC* mutant construction
ybgC-delta3	accgggatgtaaagtgaatacaattgtcgcggagtttaagcag	*ybgC* mutant construction
ybgC-delta4	gcggtcgacccaaacagaccaatatacgg	*ybgC* mutant construction
ybgF-delta1	gcgggatccagcaatgacggcagcgaagg	*ybgF* mutant construction
ybgF-delta2	tcgtgtgttatgcattacatcgcgttactgctcatgcaattctc	*ybgF* mutant construction
ybgF-delta3	aagagaattgcatgagcagtaacgcgatgtaatgcataacacac	*ybgF* mutant construction
ybgF-delta4	gcggtcgacgaacccacagtaattagtgg	*ybgF* mutant construction
fliC-delta1	gcgggatccagtattggcggtctggaaag	*fliC* mutant construction
fliC-delta2	caggttacggcgattaaccctgttgtgccatgattcgttatcc	*fliC* mutant construction
fliC-delta3	ggataacgaatcatggcacaacagggttaatcgccgtaacc	*fliC* mutant construction
fliC-delta4	agcccgggagcagatcgtcaagttccac	*fliC* mutant construction
motA-delta1	gcgggatcctggcttgtgtaatggcgtcg	*motA* mutant construction
motA-delta2	tgcggatttttcaccgcacgcacgataagcacgacatcatcc	*motA* mutant construction
motA-delta3	ggaaggatgatgtcgtgcttatcgtgcgtgcggtgaaaaatccg	*motA* mutant construction
motA-delta4	gcggtcgaccgtaacgcccgcagtttcgg	*motA* mutant construction
ompA-delta1	gcgggatcctttgactgcagaagagcatgc	*ompA* mutant construction
ompA-delta2	ccagacgagaacttaagcctgctttttcattttttgcgcctcg	*ompA* mutant construction
ompA-delta3	cgaggcgcaaaaaatgaaaaagcaggcttaagttctcgtctgg	*ompA* mutant construction
ompA-delta4	gcggtcgacagcggttggaaatggaagtatc	*ompA* mutant construction
pTrc-tolB-F	gcgccatggtgatgaagcaggcattacgtc	pTrc99KtolB construction
pTrc-tolB-R	gcggtcgactcacagatacggcgaccag	pTrc99KtolB construction
pTrc-fliC-F	gcgccatggcacaagtcattaatacc	pTrc99KfliC construction
pTrc-fliC-R	gcggtcgacttaaccctgcagcagagac	pTrc99KfliC construction
pTrc-motA-F	gcgccatggtgcttatcttattaggttacc	pTrc99KmotA construction
pTrc-motA-R	gcgggatcctcatgcttcctcggttgtcg	pTrc99KmotA construction
rrsA-qPCR-F	cggtggagcatgtggtttaa	Quantitative real-time PCR
rrsA-qPCR-R	gaaaacttccgtggatgtcaaga	Quantitative real-time PCR
rpoD-qPCR-F	caagccgtggtcggaaaa	Quantitative real-time PCR
rpoD-qPCR-R	gggcgcgatgcacttct	Quantitative real-time PCR
flhD-qPCR-F	gacaacgttagcggcactga	Quantitative real-time PCR
flhD-qPCR-R	ttgattggtttctgccagctt	Quantitative real-time PCR
fliC-qPCR-F	tccactgaaagctctggatgaa	Quantitative real-time PCR
fliC-qPCR-R	cccagggatgaacggaatt	Quantitative real-time PCR
fliA-qPCR-F	cgagcgtggaacttgacgat	Quantitative real-time PCR
fliA-qPCR-R	cgacggcattaagtaacccaat	Quantitative real-time PCR
motA-qPCR-F	tgcagggattgggtcgtt	Quantitative real-time PCR
motA-qPCR-R	cgtgcctttaatcgctttgc	Quantitative real-time PCR

To construct the IPTG-inducible *tolB*, *fliC*, and *motA* expression plasmids pTrc99KtolB pTrc99KfliC and pTrc99KmotA, we, respectively, PCR-amplified these genes with the primer pairs shown in Table 2. These products were digested with *Nco*I and *Sal*I for pTrc99KtolB and pTrc99KfliC construction or *Nco*I and *Bam*HI for pTrc99KmotA construction and ligated into similarly digested pTrc99K plasmids (Hirakawa et al., 2003). All constructs were confirmed by DNA sequencing.

### Static Biofilm Assays

Bacterial adhesion and aggregation on a 96-well polystyrene plate was assessed by a static biofilm assay using crystal violet as described elsewhere, with slight modifications (O’Toole, 2011). Bacteria were cultured for 24 h at 37°C in LB medium without shaking. Each culture was diluted into RPMI1640 medium at a 1:100 ratio, and 2.4 × 10^4^ cells/well were seeded into a 96-well polystyrene plate in triplicate. The plate was then incubated at 37°C under an atmosphere of 5% CO_2_. Cells attached to the plate were stained with crystal violet and absorbance at 595 nm (*A*_595_) was measured for each. The capability for adhesion and aggregation was determined as the *A*_595_ normalized to an OD_600_ of 1.

### Bacterial Cell Imaging by Confocal Microscopy

Imaging of bacterial cells attached to glass coverslips using the SYTO-9 staining method was performed as described previously (Kurabayashi et al., 2016). Bacteria were cultured on glass coverslips in 6-well polystyrene plates at 37°C under an atmosphere of 5% CO_2_ in RPMI1640 medium. After removal of planktonic cells by washing with phosphate-buffered saline (PBS), bacterial cells were stained with 5 μM of SYTO-9 (Life technologies, Carlsbad, CA, United States). Fluorescent images were acquired using the Alexa Fluor 488 laser module on an Olympus FV1200 IX81 microscope using a 60× objective and captured with a CCD camera.

## Infection of Bladder Epithelial Cells

Bacterial cell adhesion to, and internalized into the host epithelial cells were assessed by gentamicin protection assays as described previously (Kurabayashi et al., 2016). HTB-9 cells were cultured to confluence in 24-well plates and then inoculated with a multiplicity of infection (m.o.i) of 10 bacteria per host cell in triplicate wells. After infection for 2 h, the total number of bacteria was determined from a first set of triplicate wells. The number of adhered/internalized bacteria was determined by counting the number of bacteria present in a second set of wells after washing five times with PBS + (PBS containing 0.5 mM MgCl_2_ and 1 mM CaCl_2_). The number of internalized bacteria only was determined by counting the number of bacteria present in a third set of wells after gentamicin treatment for further 2 h. Numbers of adhered/internalized and internalized bacterial cells are represented as relative CFUs (colony forming units) by their ratios (%) to total cell CFUs.

We also imaged bacterial cells in HTB-9 cells using confocal microscopy as described previously (Kurabayashi et al., 2016). A UPEC strain carrying a green fluorescence protein (GFP) expression plasmid, pTurboGFP-B (Evrogen, Moscow, Russia) was inoculated with a multiplicity of infection (m.o.i) of 10 bacteria per host cell into cultured HTB-9 cells on glass coverslips in a 6-well plate and incubated for 2 h at 37°C under an atmosphere of 5% CO_2_. Non-internalized bacteria were washed out by gentamicin and PBS+. The HTB-9 cells were stained with rhodamine-phalloidin (Life technologies, Carlsbad, CA, United States). Fluorescent images were acquired using an Olympus FV1200 IX81 microscope with 60x objective and a CCD camera.

### Urinary Tract Infections in Mice

Six- to seven- week old C3H/HeN female mice were obtained from Japan SLC, Inc., (Hamamatsu, Japan). The mice were fed up to eight-week old and then deprived of water for 18 h before infection to minimize the possibility that bacteria being flushed out with urine. Twenty-four-hour static cultures of the UPEC were harvested and re-suspended in PBS at a concentration of 2 × 10^9^ CFU/mL. Mice were anesthetized and infected via transurethral catheterization using polyethylene tubing (inner diameter 0.28 mm and outer diameter 0.61 mm) (Becton Dickinson, Franklin Lakes, NJ, United States) using 50 μL of the bacterial suspension (1 × 10^8^ CFU). Water deprivation was continued for another 4 h following bacterial injection. Mice were euthanized 48 h post-infection and their bladders and kidneys were aseptically removed and homogenized in PBS. Diluted suspensions were plated on LB agar to determine bacterial CFUs. Data were analyzed using GraphPad Prism version 6.00, with statistical significance, considered a *P* value of <0.05, being determined using Mann-Whitney tests. All animal studies were approved by the Animal Research Committee of Gunma University.

### Hemagglutination (HA) Assays

To estimate the activity of type 1 fimbria, we tested the hemagglutination of guinea pig red blood cells (RBCs). Bacteria were statically cultured in RPMI1640 medium at 37°C under an atmosphere of 5% CO_2_ and, then harvested in duplicate tubes. The cell pellets in a first tube were re-suspended in PBS while those in the other tube were re-suspended in PBS containing 1% of mannose. Then, both tubes were incubated for 10 min at room temperature. These suspensions were serially diluted in 96-well round bottom plates containing PBS and an equal volume of 1% RBC suspension was then added. We also measured the activity of P-type fimbriae by testing the hemagglutination of RBCs extracted from human O-type blood. After incubation for 4 h at 4°C, HA titers were determined. The titers are presented as the reciprocal of the dilution of the last well before erythrocyte buttons were formed.

### Motility Assays

RPMI1640 medium containing 0.3% agar were spotted with 2 μL of bacteria grown for 24 h at 37°C in LB medium. Bacterial motility was evaluated by measuring the motility diameters after 10 h at 37°C under an atmosphere of 5% CO_2_.

### Flagellum Staining

Bacteria were cultured for 24 h at 30°C in heart infusion (HI) medium containing 0.3% agar. Two colonies were re-suspended in PBS, and streaked on a slide glass. After flame fixation, flagella were stained with Victoria blue/Tannic acid solution (0.2 *g* Victoria blue B, 9.1 *g* Tannic acid, 2.3 *g* potassium alum sulfate, 2.3 mL phenol, 6.9 mL 2-propanol and 90 mL distilled water) for 7 min.

### RNA Extraction and Quantitative Real-Time PCR Analyses

Bacteria were cultured for 24 h at 37°C in LB medium without shaking. Each culture was diluted into RPMI1640 medium at a 1:100 ratio and incubated for 4 h (to late-logarithmic growth phase; OD_600_∼0.5). Total RNA extraction and cDNA synthesis were carried out using an SV Total-RNA Isolation System and GoScript^TM^ Reverse Transcription System by following the manufacturer’s instructions (Promega Corp., Madison, WI, United States). Real-time PCR included 2.5 ng cDNA and 200 nM primers (listed in Table 2) in SYBR Select Master Mix (Applied Biosystems, Foster City, CA, United States) and were run on an ABI Prism 7900HT Fast Real Time PCR System. The constitutively expressed *rrsA* and *rpoD* genes were used as internal controls.

### Analysis of Outer Membrane Proteins

Bacteria were grown to late-logarithmic growth phase at 37°C. Cells were harvested and stored at −80°C overnight. The cell pellet was suspended in phosphate buffer (50 mM sodium phosphate [pH 7.0]) and lysed by sonication on QSonica sonicator by following the manufacturer’s instructions (Qsonica L.L.C, Newtown, CT, United States). The lysates were centrifuged for 5 min at 12,000 *g* to remove unbroken cells. The supernatant fractions were centrifuged for 40 min at 210,000 *g*. To solubilize inner membranes, the pellets were re-suspended in phosphate buffer (20 mM sodium phosphate [pH 7.0]) containing 2% *N*-lauroylsarcosine, and incubated with rolling for 60 min (Beis et al., 2006). The extracts were centrifuged again for 60 min at 210,000 *g*. The resulting pellets contain enriched outer membranes, and were solubilized in Laemmli sample buffer (Bio-Rad Laboratories, Hercules, CA, United States). The proteins localized in outer membrane were separated by SDS-PAGE on a 10% acrylamide gel, and stained with Coomassie brilliant blue.

## Results

### Deletion of the *tolB* Gene Decreases Attachment and/or Aggregation to Polystyrene and Glass Surfaces

**FIGURE 1:**
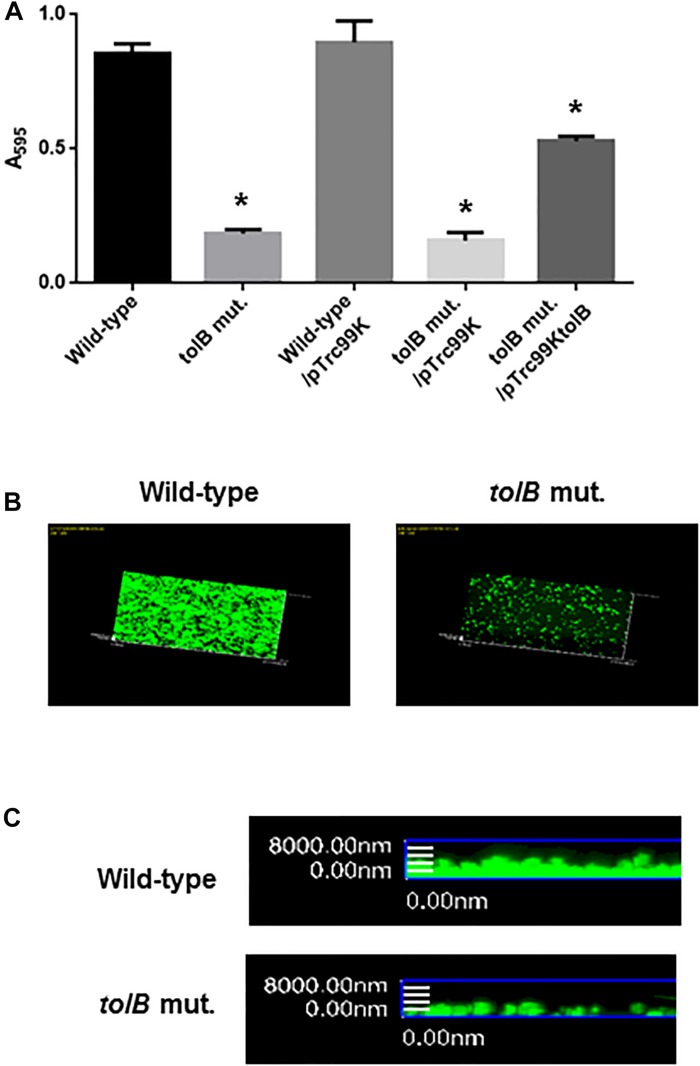
Bacterial adhesion and aggregation on 96-well polystyrene plates in the wild-type parent and the *tolB* mutant, or the wild-type parent and the *tolB* mutant carrying pTrc99K (empty vector) or pTrc99KtolB (*tolB* expression plasmid) **(A)**. Bacterial adhesion and aggregation were represented as A_595_ values normalized to OD_600_ of 1. Data plotted are the means of three biological replicates, error bars indicate the ranges, ^*^P < 0.01. Asterisks denote significance for values relative to the wild-type control. Attachment of the wild-type parent and the *tolB* mutant on cover glasses. Bacteria stained with SYTO-9 were imaged as a green fluorescent color on the microscopy using 60× objective. Overlooking **(B)** and cross sectional **(C)** images were acquired for each sample. The experiment was repeated twice and similar results were obtained.

We identified a clone in a transposon library that we constructed in our previous work, that had a transposon inserted into its *tolB* gene and that exhibited a lower degree of attachment to 96-well polystyrene plates than the wild-type parent when cultured in LB medium. We confirmed this effect by testing an in-frame deletion mutant of the *tolB* gene (CFT073ΔtolB). This strain also attached to plates less effectively than its wild-type parent. We also tested the mutant after culture in RPMI1640 medium that is used for growing bladder epithelial cells. Similarly to when cultured in LB medium, the mutant adhered less than its wild-type parent (Figure 1A). Cell attachment of CFT073ΔtolB was restored to approximately 70% of the wild-type level by the introduction of pTrc99KtolB, an IPTG-inducible heterologous *tolB* expression plasmid (Figure 1A).

In addition to assays using 96-well polystyrene plates, *tolB* mutant or the wild-type bacterial cells was attached to glass coverslips and imaged by fluorescence confocal microscopy after staining with SYTO-9. The CFT073ΔtolB cells attached to a lower extent compared with the wild-type (Figures 1B,C). These observations suggest that the *tolB* gene contributes to attachment and/or aggregation of UPEC.

### The *tolB* Gene Is Required for Optimal Internalization Into and Aggregation Within Bladder Epithelial Cells, but Not for Adhesion

**FIGURE 2:**
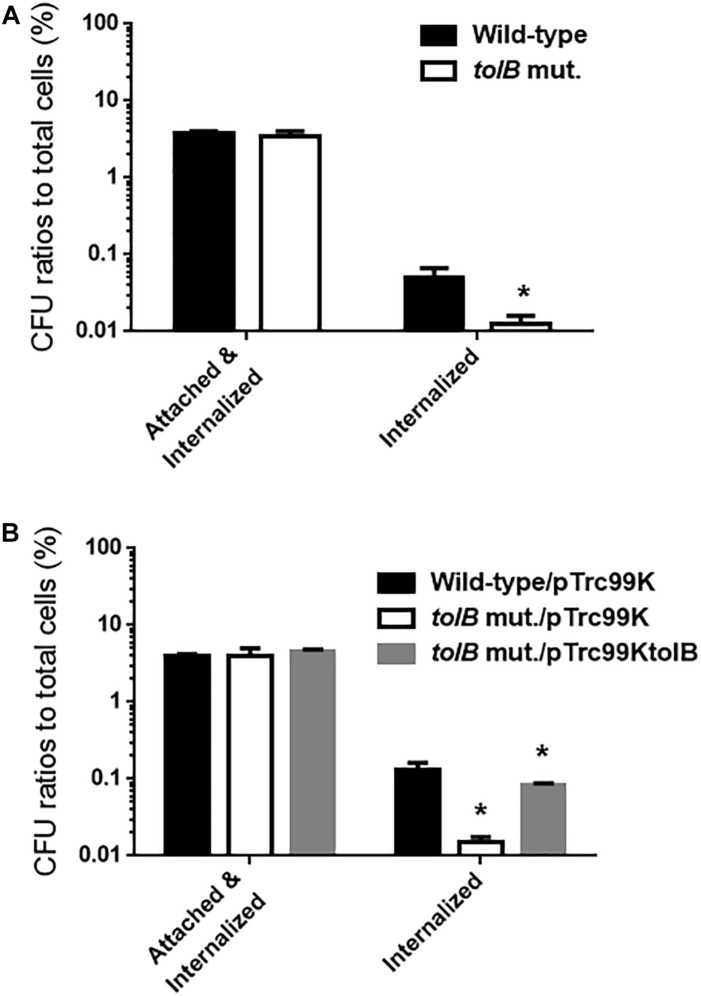
Adhesion to and internalization of bladder epithelial cells (HTB-9) for the wild-type parent and the *tolB* mutant **(A)** or the wild-type parent and the *tolB* mutant carrying pTrc99K (empty vector) or pTrc99KtolB (*tolB* expression plasmid) **(B)**. *Y*-axis on the graphs shows percent (%) of CFU (colony forming units) values of adhered and internalized bacteria relative to total bacterial cell numbers. Data plotted are the means from three independent experiments; error bars indicate the standard deviations, ^*^P < 0.01. Asterisks denote significance for values relative to the wild-type control.

The reduced bacterial mass on polystyrene and glass surfaces induced by *tolB* gene deletion may reflect a reduction of adhesion to, internalization into, and aggregation within bladder epithelial cells. To test this hypothesis, we compared levels of UPEC cell adherence and internalization between the *tolB* mutant and its wild-type parent after inoculation onto bladder epithelial cells using a gentamicin protection assay. The total adherence/internalization of the *tolB* mutant was essentially the same as for the wild-type parent (3.80 ± 0.16% for the wild-type parent and 3.45 ± 0.43% for the *tolB* mutant). However, the *tolB* mutant exhibited an approximately 4-fold reduced degree of internalization alone (0.051 ± 0.011% for the wild-type parent and 0.013 ± 0.002% for the *tolB* mutant) (Figure 2A). We also confirmed that the internalization of the *tolB* mutant was significantly elevated by introducing the pTrc99KtolB plasmid (internalization rate; 0.13 ± 0.02% for the wild-type parent carrying the pTrc99K, empty vector, 0.015 ± 0.002% for the *tolB* mutant carrying pTrc99K and 0.083 ± 0.004% for the *tolB* mutant carrying pTrc99KtolB) (Figure 2B).

**FIGURE 3:**
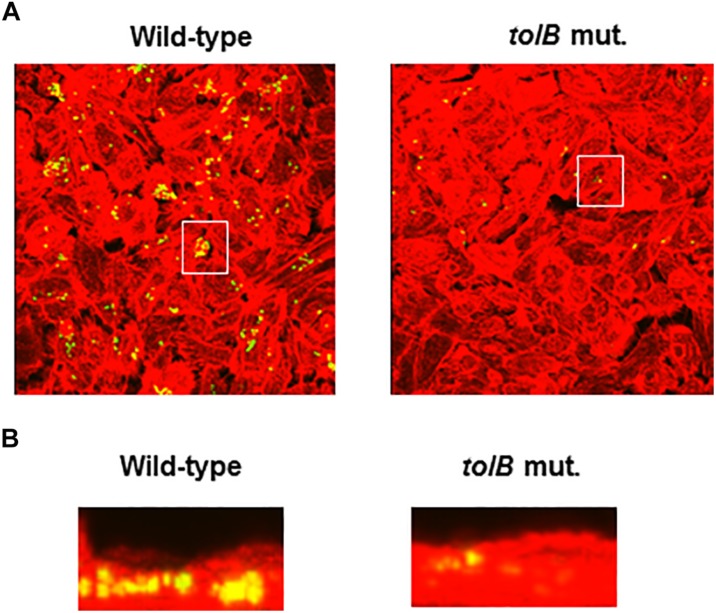
Fluorescent images of aggregated UPEC cells within bladder epithelial cells (HTB-9). Bacteria carrying a Green fluorescence protein (GFP) expression plasmid, pTurboGFP-B and HTB-9 stained with rhodamine- phalloidin were imaged as, respectively, green and red fluorescent colors on the microscopy using 60× objective. Overlooking images **(A)** and cross sectional images in the white box indicated in panel **A** **(B)** were acquired for each sample. The experiment was repeated twice and similar results were obtained.

To compare colonies of the *tolB* mutant with the wild-type parent inside bladder epithelial cells, we infected host cells with the wild-type parent or *tolB* mutant (both containing a GFP expression plasmid), and then imaged the bacteria and cellular actin. The wild-type parent produced bacterial colonies inside the epithelial cells while those formed by the *tolB* mutant were relatively small and less frequent (Figures 3A,B). We note that a complementation test by introducing pTrc99KtolB into the mutant was not performed in this case. Unfortunately, the pTrc99K plasmid is not compatible with the pTurbo-GFP-B plasmid because both plasmids have same replicon. However, results of this microscopy experiment were supported by gentamicin protection assays. In addition, we repeated this experiment, then results were highly reproducible.

Considering results of these bladder epithelial cells experiments, the reduced bacterial mass on polystyrene and glass surfaces in *tolB* mutant could be due to a defect in bacterial aggregation but not adhesion. Thus, *tolB* gene is not required for adhesion, but is for optimal internalization into, and aggregation within bladder epithelial cells.

### The *tolB* Gene Is Responsible for Colonization of UPEC in the Murine Urinary Tract

**FIGURE 4:**
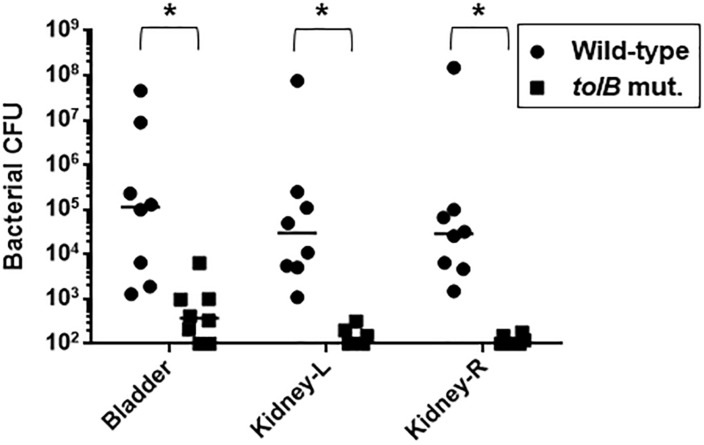
Colonization of the wild-type and *tolB* mutant in bladder and kidneys of UTI mice. At 48 h of post infection, bladder, left kidney (kidney-L) and right kidney (kidney-R) were aseptically and separately removed. Each cell number of bacteria isolated from these organs was represented by CFU (colony forming unit). Each data point represents a sample from an individual mouse. Horizontal bars indicate the median values. The *tolB* mutant was colonized in both bladder and kidneys in significantly lower degree than the wild-type parent (*P* < 0.01). Asterisks denote significance for values.

To investigate the role of *tolB* in the pathogenesis of UPEC in the murine urinary tract, 1 × 10^8^ CFU of either the *tolB* mutant or the wild-type parent were inoculated into C3H/HeN mice via a urethral catheter, and the number of bacteria in the bladder and kidneys were determined 48 h after inoculation. There were fewer *tolB* mutant in these organs than those of the wild-type parent (median of CFU values; bladder: 1.2 × 10^5^ for the wild-type parent and 3.7 × 10^2^ for the *tolB* mutant, left kidney: 3.1 × 10^4^ for the wild-type parent and <1.0 × 10^2^ for the *tolB* mutant, right kidney: 2.9 × 10^4^ for the wild-type parent and <1.0 × 10^2^ for the *tolB* mutant) (Figure 4). As with the case of our microscopy experiment, the complementation test was not performed in this mice experiment because it is still unclear whether the pTrc99K plasmid works in mice although this system worked well for *in vitro* experiments by exogenously adding IPTG inducer into media. To confirm the phenotype of the *tolB* mutant in the murine UTI model, we repeated this experiment, then we obtained similar result.

We also note that there was no significant difference in the growth of these strains when cultured in AUM or RPMI1640 (data not shown) therefore, the reduction of bacterial numbers in the mouse bladder and kidneys in the *tolB* mutant was unlikely to be solely due to a growth defect.

### Deletion of the *tolB* Gene Affects Neither Activity nor Expression of Type 1 and P-Type Fimbriae

Type 1 fimbriae are the most major bacterial components required for development of UTIs following adhesion and internalization (Lillington et al., 2014). FimH, the fimbrial-tip adhesin, mediates interaction with highly mannosylated transmembrane proteins such as uroplakins on urothelium cells (Hultgren et al., 1986). It also mediates agglutination of guinea pig erythrocytes, however, the agglutination is inhibited by the addition of mannose, blocking the interaction between FimH and the mannosylated receptor on the erythrocytes (Hagberg et al., 1981). Therefore, we examined the effect of *tolB* deletion on type 1 fimbrial activity by agglutination titers between the wild-type parent and *tolB* mutant using guinea pig erythrocytes. Unexpectedly, the mutant cells exhibited a two-fold higher agglutination titer than the wild-type parent did when they were re-suspended in mannose-free PBS (Titers: 32 for the wild type parent and 64 for the *tolB* mutant), while in the presence of mannose, the titers for both strains were only 1 (Table 3).

**TABLE 3:** HA titers of the wild-type parent and the *tolB* mutant.

	**HA titers for type 1 fimbrial activities**	
**Strain**	**− Mannose**	**+ Mannose**
Wild-type (CFT073)	32	1
*tolB* mutant (CFT073ΔtolB)	64	1

**Strain**	**HA titers for P-type fimbrial activities**	

Wild-type (CFT073)	16	
*tolB* mutant (CFT073Δtol)B	16	

We also estimated the activities of P-type fimbriae by comparing agglutination titers between the wild-type parent and *tolB* mutant using human type O erythrocytes, because P-type fimbriae are the second-most common fimbriae that UPEC CFT073 produces, and play a role in the pathogenesis of ascending UTIs and pyelonephritis in humans (Hagberg et al., 1981; Lane and Mobley, 2007). The agglutination titers of human type O erythrocytes were same between the wild-type parent and *tolB* mutant cells (16 for both strains) (Table 3).

### Deletion of the *tolB* Gene Decreases Mature Flagellar Production, Resulting in a Reduction in Motility

**FIGURE 5:**
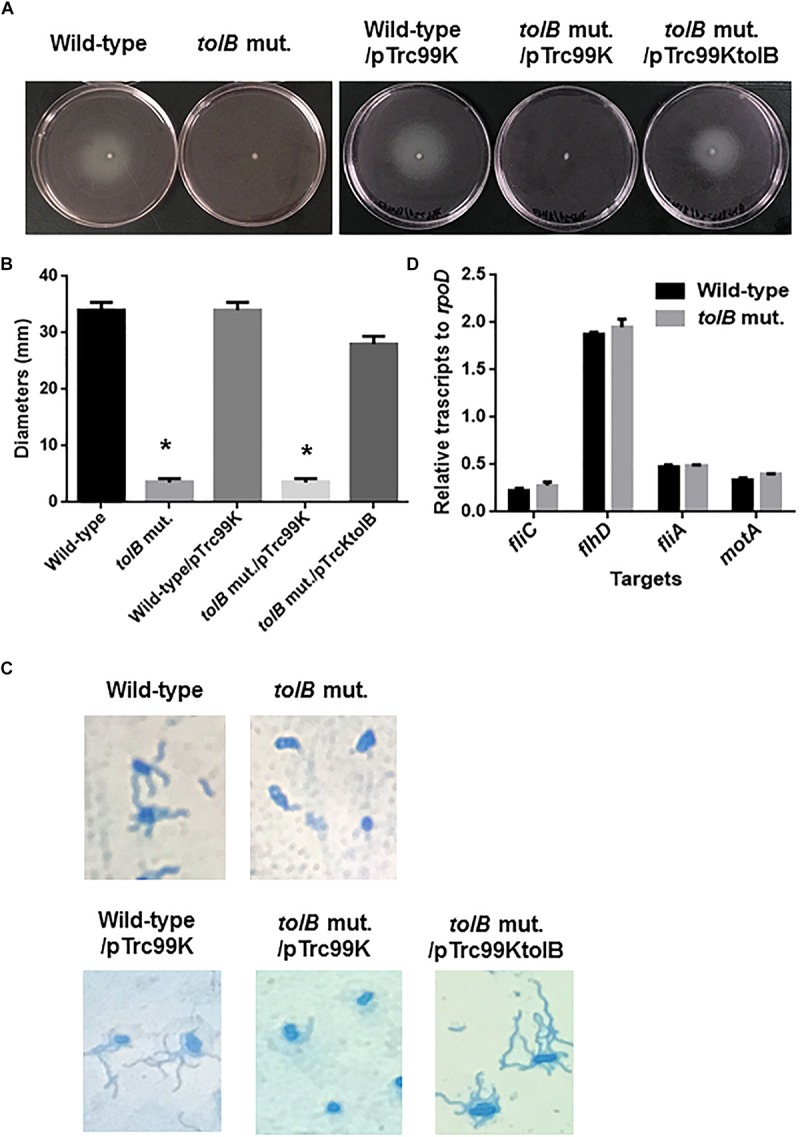
Motilities of the wild-type parent and *tolB* mutant, or wild-type parent and *tolB* mutant carrying pTrc99K (empty vector) or pTrc99KtolB (*tolB* expression plasmid). **(A)** Bacterial migrations on RPMI1640 medium containing 0.3% agar after 10 h at 37°C under 5% of CO_2_ were pictured. **(B)** Bacterial migrations on the agar were represented as diameters. Data plotted are the means from three independent experiments; error bars indicate the standard deviations ^*^P < 0.01. Asterisks denote significance for values relative to the wild-type control. **(C)** Flagella and bacteria cells stained with Victoria blue/Tannic acid were pictured on the microscopy using 100× objective. **(D)** Transcript levels of motility-related genes in the wild-type parent and the *tolB* mutant. Transcript levels were described as relative values to that of *rpoD* (housekeeping gene). Data plotted are the means of two biological replicates, error bars indicate the ranges.

**FIGURE 6:**
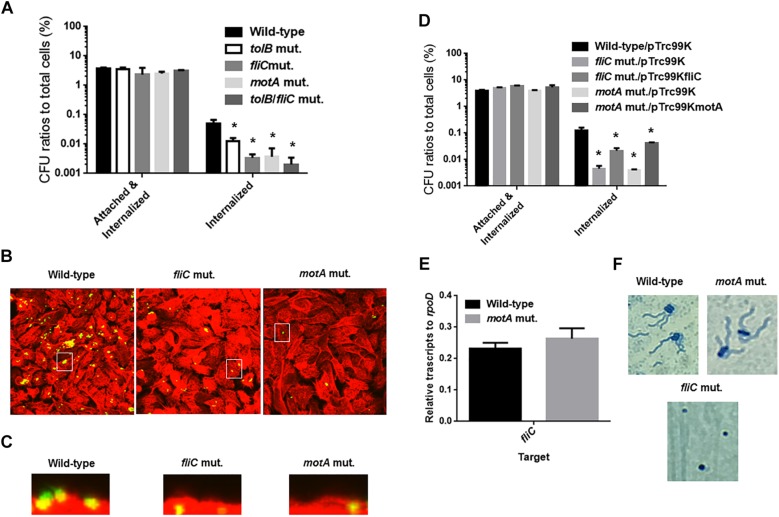
Adhesion to, internalization of and aggregation within bladder epithelial cells (HTB-9) for the wild-type parent, and the *tolB*, *fliC*, and *motA* mutants, or wild-type parent and mutants carrying pTrc99K (empty vector), pTrc99KfliC (*fliC* expression plasmid) or pTrc99KmotA (*motA* expression plasmid). *Y*-axis on the graphs shows percent (%) of CFU (colony forming units) values of adhered and internalized bacteria relative to total bacterial cell numbers **(A,D)**. Data plotted are the means from three independent experiments; error bars indicate the standard deviations ^*^P < 0.01. Asterisks denote significance for values relative to the wild-type control. Fluorescent images of aggregated UPEC cells within bladder epithelial cells (HTB-9). Bacteria carrying a Green fluorescence protein (GFP) expression plasmid, pTurboGFP-B and HTB-9 stained with rhodamine-phalloidin were imaged as, respectively, green and red fluorescent colors on the microscopy using 60× objective. Overlooking images **(B)** and cross sectional images in the white box indicated in panel **B** **(C)** were acquired for each sample. The experiment was repeated twice and similar results were obtained. Transcript levels of *fliC* gene in the wild-type parent and the *tolB* mutant **(E)**. Transcript levels were described as relative values to that of *rpoD* (housekeeping gene). Data plotted are the means of two biological replicates, error bars indicate the ranges. Flagella and bacteria cells stained with Victoria blue/Tannic acid were pictured on the microscopy using 100× objective **(F)**.

Flagella are necessary for migration of UPEC toward epithelial cell surfaces in the urinary tract, and also provide fitness at urinary infection sites (Lane et al., 2005; Wright et al., 2005). Decreased internalization and aggregation in the *tolB* mutant may be due to a defect in motility and/or flagellar production. To test this possibility, we compared the motilities of the wild-type parent and *tolB* mutant on soft agar pates and found that the mutant exhibited a lower motility than the wild-type parent (Diameters: 34 ± 1 mm for the wild type parent and 3 ± 1 mm for the *tolB* mutant) (Figures 5A,B). The data of flagellum stain experiment suggested the impaired flagellar production in *tolB* mutant although mRNA levels of *flhD* (which encodes the activator for flagellar expression), *fliC* (which encodes a major flagellar component) and *fliA* (which encodes a flagellar biosynthesis sigma factor) in the *tolB* mutant were similar to those in the wild-type parent (Figures 5C,D). We also found that deletion of *tolB* does not affect the expression of *motA* (which encodes a motor protein for flagellar rotation) (Figure 5D). When a plasmid containing the *tolB* gene was introduced into the mutant, its motility increased to the level of the wild-type parent (Diameters: 34 ± 1 mm for the wild type parent carrying pTrc99K, 3 ± 1 mm for the *tolB* mutant carrying pTrc99K and 28 ± 1 mm for the *tolB* mutant carrying pTrc99KtolB) (Figures 5A,B). Additionally, the flagella of the mutant carrying pTrc99KtolB were similar to the wild-type (Figure 5C). We confirmed that UPEC motility is indeed important for internalization and aggregation by using *fliC* and *motA* mutants. The *fliC* and *motA* genes encode flagellin and motor protein for flagellar rotation, respectively. These mutants exhibited lower rates of bacterial internalization into, and aggregation within bladder epithelial cells compared to the wild-type parent (0.051 ± 0.011% for the wild-type parent, 0.0034 ± 0.0007% for the *fliC* mutant and 0.0037 ± 0.0024% for the *motA* mutant), while their adhesion were similar to the wild-type parent (Figures 6A–C). We also constructed a *tolB*/*fliC* double-mutant and found that its internalization was similar to that of the *fliC* single mutant (0.0021 ± 0.0011 for the *tolB*/*fliC* double-mutant and 0.0034 ± 0.0007% for the *fliC* single-mutant). We also confirmed that bacterial internalization in *fliC* and *motA* mutants was promoted by introducing the complementary expression plasmids, pTrc99KfliC and pTrc99KmotA, respectively (0.0046 ± 0.0008% for the *fliC* mutant carrying pTrc99K and 0.022 ± 0.003% for the *fliC* mutant carrying pTrc99KfliC, and 0.0040 ± 0.0002% for the *motA* mutant carrying pTrc99K and 0.043 ± 0.002% for the *motA* mutant carrying pTrc99KmotA) (Figure 6D). We confirmed that the *motA* mutant produces flagella at similar level to the wild-type parent (Figures 6E,F). These results suggest that defective motility of the *tolB* mutant is involved in the decreased levels of bacterial internalization and aggregation.

### Deletion of *tolA*, *tolQ*, *tolR*, and *pal*, but Not *ybgC* and *ybgF*, Which Encode Proteins That Together Work With TolB in the Tol-Pal System Decreases Motility and Levels of Bacterial Aggregation Within Bladder Epithelial Cells

**FIGURE 7:**
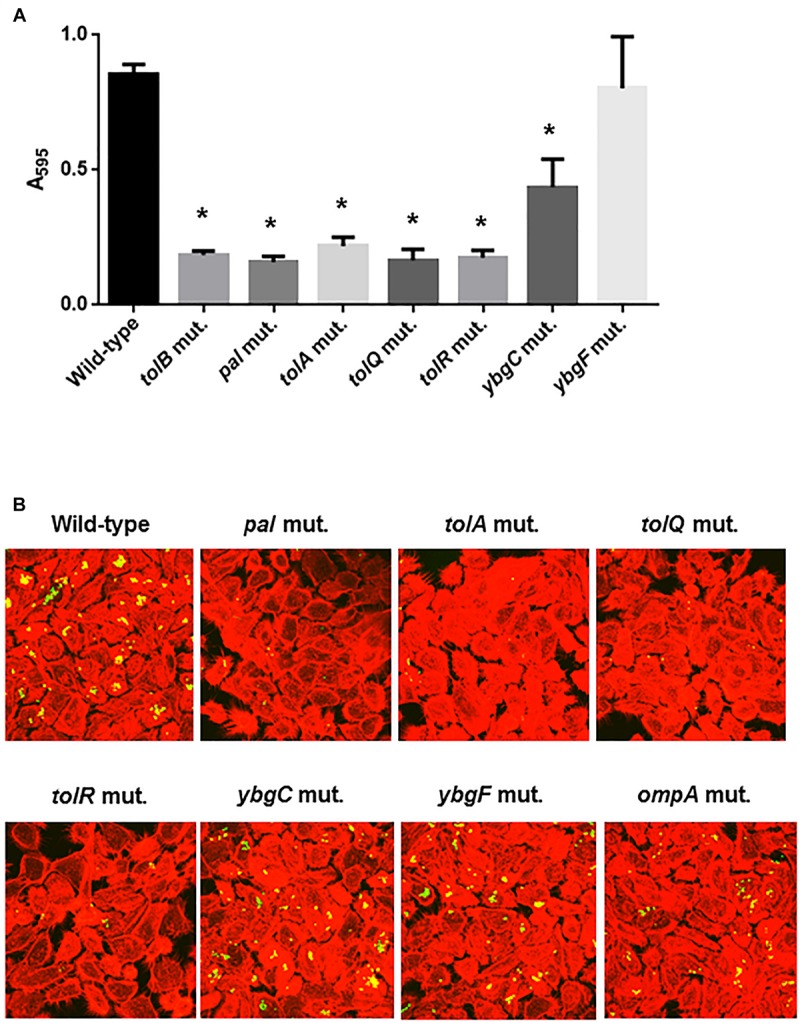
Bacterial adhesion and aggregation on 96-well polystyrene plates in the wild-type parent, and the *tol*-*pal*, *ybgC*, *ybgF*, and *ompA* mutants **(A)**. Bacterial adhesion and aggregation were represented as A_595_ values normalized to OD_600_ of 1. Data plotted are the means of three biological replicates, error bars indicate the ranges, ^*^P < 0.01. Asterisks denote significance for values relative to the wild-type control. Fluorescent images of aggregated UPEC cells within bladder epithelial cells (HTB-9) **(B)**. Bacteria carrying a Green fluorescence protein (GFP) expression plasmid, pTurboGFP-B and HTB-9 stained with rhodamine-phalloidin were imaged as, respectively, green and red fluorescent colors on the microscopy using 60× objective. The experiment was repeated twice and similar results were obtained.

**FIGURE 8:**
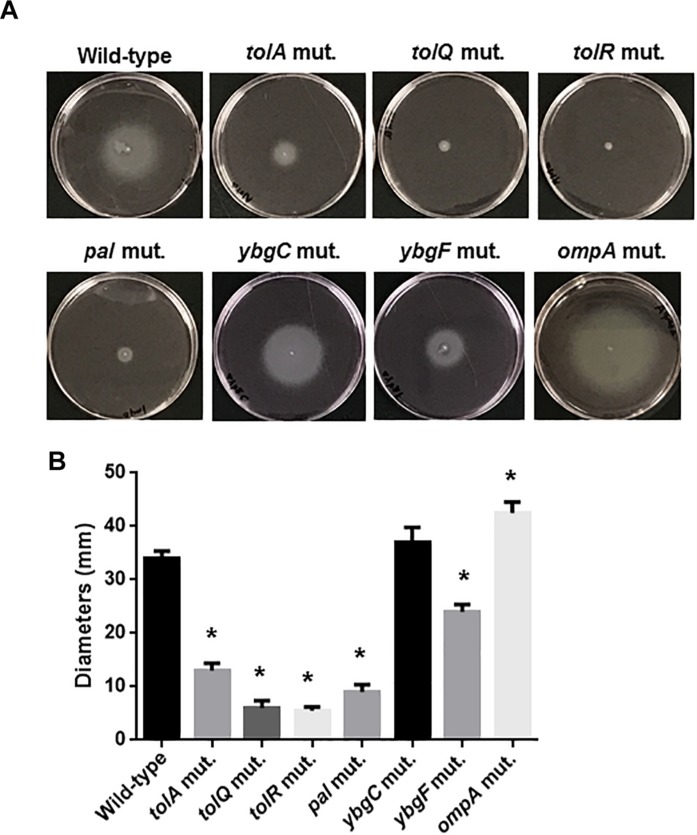
Motilities of the wild-type parent, and the *tol*-*pal* and *ybgC*, *ybgF*, and *ompA* mutants. **(A)** Bacterial migrations on RPMI1640 medium containing 0.3% agar after 10 h at 37°C under 5% of CO_2_ were pictured. **(B)** Bacterial migrations on the agar were represented as diameters. Data plotted are the means from three independent experiments; error bars indicate the standard deviations ^*^P < 0.01. Asterisks denote significance for values relative to the wild-type control.

The *tolB* gene product forms protein complexes with the TolA, TolQ, TolR, and Pal proteins, and together these constitute the Tol-Pal system (Walburger et al., 2002). Other genes, *ybgC* and *ybgF* are transcribed with *tolQ*, *tolR*, *tolA*, and *tolB*, and *pal*, from two separate operons although their roles have not been fully elucidated (Vianney et al., 1996; Muller and Webster, 1997). We tested whether these genes are also involved in motility-associated internalization and aggregation. Static biofilm assays showed that the levels of bacteria attached to 96-well polystyrene plates in the *tolQ*, *tolR*, *tolA* and *pal* mutants were similar of those for the *tolB* mutant, while the *ybgF* mutant attached at the same levels as the wild-type parent (Figure 7A). The attachment level of the *ybgC* mutant was approximately 50% lower than that of the wild-type parent and the *ybgF* mutant, however, it is still significantly higher than that of the *tolB* mutant (Figure 7A). We compared bacterial colonies of these strains carrying a GFP expression plasmid after inoculation into bladder epithelial cells. As with the *tolB* mutant, bacterial colonies formed by the *tolQ*, *tolR*, *tolA*, and *pal* mutants were smaller and less frequent than the wild-type parent (Figure 7B). In contrast, the *ybgC* and *ybgF* mutants produced colonies that were indistinguishable from the wild-type parent (Figure 7B). Consistent with colony formation, the *tolQ*, *tolR*, *tolA*, and *pal* mutants were less motile than the wild-type parent and the *ybgC* and *ybgF* mutants (Diameters: 34 ± 1 mm for the wild type parent, 11 ± 2 mm for the *tolA* mutant, 6 ± 1 mm for the *tolQ* mutant, 5 ± 1 mm for the *tolR* mutant, 9 ± 1 mm for the *pal* mutant, 37 ± 2 mm for the *ybgC* mutant and 24 ± 1 mm for the *ybgF* mutant) (Figures 8A,B). These results indicate that a subset of genes required for the Tol-Pal system contribute to UPEC motility and its aggregation within bladder epithelial cells.

### The *ompA* Gene Is Not Required for Optimal Motility or Internalization Into, and Aggregation Within Bladder Epithelial Cells

The *tolB* gene product interacts with OmpA, an outer-membrane localized protein, therefore deletion of the *tolB* gene might perturb the localization and function of OmpA and, therefore, outer membrane integrity (Clavel et al., 1998). One study showed that deletion of *ompA* impairs chronic colony formation in UTI mice, but does not affect internalization of *ex vivo*-cultured bladder epithelium which is used as a model of the initial stage of UTI (Nicholson et al., 2009). Therefore, we tested whether defective motility, internalization and aggregation in the *tolB* mutant is associated with the perturbation of OmpA. Rather, the *ompA* mutant exhibited a moderate higher motility compared to the wild-type parent (Figures 8A,B) while it formed bacterial colonies at similar levels to the wild-type parent (Figure 7B). These observations suggest that OmpA does not participate in the defective motility or internalization into, and aggregation within *ex vivo* bladder epithelial cells observed in the *tolB* mutant.

### The *tolB* Mutant Exhibits a Different Protein Profile in Outer Membrane Pool From the Wild-Type Parent

**FIGURE 9:**
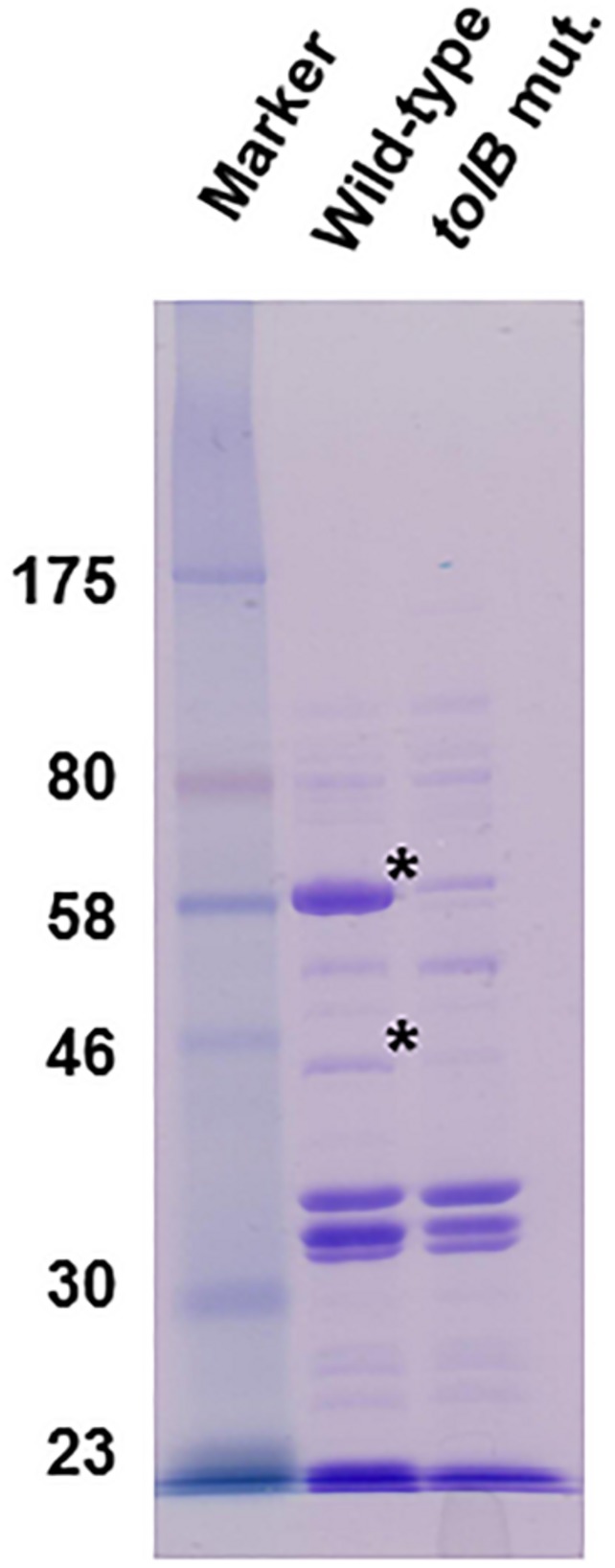
SDS-PAGE of outer membrane proteins from the wild-type parent and the *tolB* mutant. Proteins were separated on a 10% acrylamide gel, and stained with Coomassie brilliant blue. Locations of molecular mass standards (in kilodaltons) are indicated on the left. Asterisks indicate protein bands which levels are remarkably low in the *tolB* mutant compared to the wild-type parent.

TolB contributes to maintenance of the outer membrane, therefore deletion of the *tolB* gene alter the outer membrane architecture, which might globally impact expression and localization of outer membrane proteins. We analyzed a profile of outer membrane proteins in the wild-type parent and the *tolB* mutant by SDS-PAGE. We found two uncharacterized proteins corresponding to approximately 60 kilodalton (kDa) and 42 kDa which levels were remarkably lower in the *tolB* mutant than the wild-type parent (Figure 9). Thus, profile of outer membrane proteins is different between the wild-type parent and its *tolB* mutant.

## Discussion

The *tolB* gene product was originally characterized as a member of the Tol-Pal system, involved in outer membrane maintenance and uptake of colicin and filamentous phage DNA in Gram-negative bacteria (Nagel de Zwaig, 1969; Clavel et al., 1998; Webster, 1991). There are also reports that some of *tol*-*pal* genes are involved in bacterial pathogenesis. The *tolB* gene contributes to survival within macrophages and fatal infection of mice by *Salmonella enterica* serovar Typhimurium and the rot of plant leaves by the plant pathogen, *Erwinia chrysanthemi* while the *pal* gene and its product in *E. coli* and *Haemophilus ducreyi* aid in the induction of inflammation resulting in death by sepsis in mice, and pustule formation in humans, respectively (Bowe et al., 1998; Fortney et al., 2000; Hellman et al., 2002; Dubuisson et al., 2005). In this study, we showed that the *tol*-*pal* genes, including *tolB* are involved in pathogenesis by UPEC that causes UTIs. These genes are required for optimal internalization into, and aggregation within, bladder epithelial cells (Figures 1–3, 7). We also found that mutants in these genes are less motile relative to the wild-type parent (Figures 5, 8). Decreased levels of internalization into, and aggregation within, *ex vivo*-cultured bladder epithelial cells were observed in the *tolB* mutant and were associated with defective motility because ΔfliC and ΔmotA, non-motile strains also exhibited low levels of internalization and aggregation (Figure 6). Bacterial internalization into, and aggregation within, *ex vivo*-cultured bladder epithelial cells is commonly used as a model of the initial stage UTI. The *tolB* gene is also required for optimal colonization within both the bladder and kidneys (Figure 4). In other studies, a *fliC* mutant exhibited growth disadvantage when it competed with the intact-*fliC* parent strain in the urinary tracts of mice, however, the mutant still colonized at a similar rate to the parent strain in solo infection experiments (Lane et al., 2005; Wright et al., 2005). On the other hand, it has also been reported that the *ompA* gene contributes to optimal colonization in UTI mice (Nicholson et al., 2009). Tol-Pal proteins maintain the stability of OmpA, which is localized on outer membrane, therefore, deletion of the *tol*-*pal* genes, including *tolB*, presumably decreases OmpA activity. Thus, the Tol-pal proteins may be also responsible for the progression of UTI sustained by OmpA, and so the decrease in colonization of the mouse urinary tract by the *tolB* gene deletion may also involve a defect of OmpA activity. In this study, we also found two proteins from outer membrane extract of the wild-type strain although we have not yet characterized them (Figure 9). These levels were very low in the *tolB* mutant. In addition to OmpA, a possibility that any of these proteins may also contribute to UPEC pathogenicity is not excluded, therefore we try to characterize some of our interest proteins in a future project.

The defect of motility observed in *tol*-*pal* gene mutants was attributed to impaired flagellar syntheses although there was no significant difference between the wild-type parent and *tolB* mutant in the transcript levels of a gene that encodes a protein component of flagellar subunits (Figures 5, 8). In addition to UPEC, *tol*-*pal* gene mutants in *E. chrysanthemi* exhibited a reduced flagellar phenotype although it is still unknown how the *tol*-*pal* gene product aid in the synthesis of flagella (Dubuisson et al., 2005). In flagellum complex, “L-ring” is composed of FlgH embedded in the outer membrane while “rod” assembly consists of FlgB, FlgC, FlgF, and FlgG and traverses the periplasmic space and outer membrane (Schoenhals and Macnab, 1996; Zhao et al., 2013). Disturbance in the outer membrane by deletion of *tol*-*pal* genes may impair the precise localization and stabilization of the L-ring and rod assemblies, which would presumably lead to the disassembly of the flagellar basal body and/or interruption of flagellin polymerization following formation of the basal body complex containing the L-ring and rod. It is also possible that the Tol-Pal protein complex contributes to the assembly of the flagellum basal body via protein-protein interactions, and that *tol*-*pal* mutants might therefore fail to form functional flagella.

According to previous studies, the *ybgC* and *ybgF* gene products do not participate in the Tol-Pal system although they are transcribed together with the *tol*-*pal* genes (Vianney et al., 1996; Muller and Webster, 1997). Consistent with these reports, the *ybgC* and *ybgF* are also not required for internalization, aggregation and motility, which supports the contention that the Tol-Pal system, but not YbgC and YbgF, contributes to pathogenicity of UPEC during UTIs.

Flagella and motility are also involved in the virulence of not only UPEC but also other pathogenic *E. coli* species including enterohaemorrhagic *E. coli*, causing infectious diseases in the intestines. Furthermore, the Tol-Pal system is highly conserved and it has been shown that it serves multiple purposes associated with virulence in *E. coli* species (Giron et al., 2002; Haiko and Westerlund-Wikstrom, 2013). Therefore, this system could be an effective target for the treatment of infectious diseases caused by pathogenic *E. coli* species.

## Data Availability

All datasets generated for this study are included in the manuscript and/or the supplementary files.

## Ethics Statement

All animal studies were approved by the Animal Research Committee of the Gunma University.

## Author Contributions

HH, KS, and HT designed the research, analyzed the data, and wrote the manuscript. HH, KS, and KK performed the research.

## Conflict of Interest Statement

The authors declare that the research was conducted in the absence of any commercial or financial relationships that could be construed as a potential conflict of interest.
